# Genotype-phenotype correlation of HbH disease in northern Iraq

**DOI:** 10.1186/s12881-020-01141-8

**Published:** 2020-10-15

**Authors:** Rawand P. Shamoon, Ahmed K. Yassin, Ranan K. Polus, Mohamad D. Ali

**Affiliations:** 1 Department of Pathology, College of Medicine, Hawler Medical University, Erbil, Iraq; 2 Lab. Section, Thalassemia Care Center, Erbil, Iraq; 3 Department of Internal Medicine, College of Medicine, Hawler Medical University, Erbil, Iraq; 4 Department of Clinical Hematology, Nanakali Hemato-Oncology Teaching Center, Erbil, Iraq

**Keywords:** HbH disease, Genotype-phenotype correlation, Alpha-thalassaemia, Erbil, Iraq

## Abstract

**Background:**

HbH disease results from dysfunction of three, less commonly two, α-globin genes through various combinations of deletion and non-deletion mutations. Characterization of the mutations and the underlying genotypes is fundamental for proper screening and prevention of thalassaemia in any region. The aim of this study was to explore the genetic arrangements of HbH disease and to correlate the genotypes with the clinical phenotypes.

**Methods:**

A total of 44 HbH disease patients were enrolled in this study. They were clinically and haematologically assessed. The patients were tested for 21 common α-globin gene mutations using multiplex PCR and reverse hybridization. According to the genotype, the patients were categorized into two separate sub-groups, deletion and non-deletion types HbH disease.

**Results:**

Within the studied HbH disease patients, eight different α-globin gene mutations were detected in nine different genetic arrangements. The --^MED^ and -α^3.7^ deletions were the two most frequently encountered mutations (37.5 and 35.2% respectively). Patients with deletion genotypes constituted 70.4%. The most common detected genotype was --^MED^/−α^3.7^ (59.1%), followed by α^poly-A1^α/α^poly-A1^α (13.6%). For the first time, coinheritance of two relatively mild mutations (−α^3.7^/αα^Adana^) was unpredictably detected in a 1.5 year-old child with Hb of 7.1 g/dL.

**Conclusion:**

The HbH disease patients’ clinical characteristics were variable with no ample difference between the deletion and non-deletion types. These results can be of benefit for the screening and management of thalassaemia in this region.

## Background

α-thalassaemia is said to be the most common monogenic disorder worldwide. The genetic defects in the α-globin genes, which are mostly gene deletions, result in the absence or reduced production of α-globin chain in the hemoglobin tetramer [1]. Numerous mutations, deletion and non-deletion types, have been so far identified in the α-globin genes; some causes dysfunction of one α-globin gene, denoted as α^+^ mutations; others resulting in deletion or deactivation of two α-globin genes, denoted as α^0^ alleles [2]. The clinical manifestations of these genetic defects vary. In heterozygous state, these mutations are usually of little clinical significance. However, the coinheritance of these mutations may result in a variety of clinical and haematological phenotypes. The most severe phenotype is hydrops fetalis, where all four α-globin genes are missing (−−/−−). Hemoglobin H (HbH) disease is a moderate clinical form of α-thalassaemia in which three of the four α-globin genes are affected (−−/−α). The unstable HbH can precipitate in the red cells causing hemolysis and jaundice [3–5]. Less commonly, HbH disease may result from two point mutations in the α-globin genes [6–8]. Based on the nature of the genetic defects, two types of HbH disease exist. The first, and commonest, form is called deletion HbH disease that results from a large deletion mutation, which deactivates both α-globin genes on one chromosome such as --^MED^, −-^SEA^, −-^Thai^, or --^FIL^ combined with a single-gene deletion like -α^3.7^ or -α^4.2^ on the other chromosome. The second type is the non-deletion form of HbH disease, which ensues when a non-deletion mutations in either the α1 or α2 globin genes on one chromosome combines with another non-deletion mutation, or with a deletion of both α-globin genes on the other chromosome [9, 10].

α-thalassaemia mutations, which are apparently not common in this geographical location, have not been studied before, nor HbH disease genotypes were explored. This study aimed to characterize the α-globin gene mutations in this cohort of patients, discover the mutation combinations resulting in HbH disease, and correlate the disease genotypes with clinical and haematological phenotypes.

## Methods

Over 6 years, starting from February 2014, 44 cases of HbH disease were recruited to this study at Erbil Thalassaemia Care Centre, Erbil, Northern Iraq. Most of the enrolled patients were referred to the aforementioned centre from other hospitals and/or private clinics with the suspicion of having a hemoglobinopathy. All enrolled patients were interviewed and physically examined. Demographic data and disease history, specifically transfusion history, were obtained. Informed written consent was obtained from the patients. In the case of minors (less than 16 years), the guardian’s consent was obtained before obtaining their assent. The study was approved by the Research Ethics Committee of Hawler Medical University, Erbil, Iraq. The diagnosis of HbH disease was made following performing routine complete blood count, using automated haematology analyzer (Swelab, Spagna, Sweden), and peripheral blood smear; HbH inclusion test, using 1% brilliant cresyl blue staining of the red cells and incubation at 37 °C for 1 h; and measuring the HbH fraction using high performance liquid chromatography (HPLC), with the VARIANT™ HPLC system on fresh blood samples (Bio-Rad Laboratories, Hercules, CA, US) [11]. Serum ferritin level was measured with Cobas E411 analyzer, Roche®, Germany.

The molecular analysis started with DNA extraction using Gentra Puregene blood kit (Qiagen, Germantown, MD, USA). α-globin genotyping was then performed using multiplex PCR and reverse hybridization assay according to the manufacturer’s instructions (Alpha-globin StripAssay; ViennaLab Diagnostics, Vienna, Austria), covering the following 21 mutations: two single gene deletions (−α^3.7^, −α^4.2^), five double gene deletions [−-^MED^, −-^SEA^, −-^THAI^, −-^FIL^, −α^20.5^ kb], the ααα^anti-3.7^ gene triplication, two point mutations on the α1 gene [codon 14 (TGG > TAG); codon 59 (GGC > GAC) (Hb Adana)], and 11 mutations on the α2 gene [initiation codon ATG > ACG; codon 19 (GCG > GC-), IVS-I(− 5 nt) (−TGAGG); codon 59 (GGC > GAC); codon 125 (CTG > CCG) (Hb Quong Sze); Hb Constant Spring (HbCS) codon 142, Term → Gln, TAA > CAA; Hb Icaria, codon 142, Term→ Lys, TAA > AAA; Hb Pakse´, codon 142, Term→ Tyr, TAA > TAT, Hb Koya Dora, codon 142, Term→ Ser, TAA > TCA; polyadenylation signal site, poly-A1 (AATAAA>AATAAG); and poly-A2 (AATAAA>AATGAA) [12]. Based on the nature of the α-globin genetic defects, and for the sake of comparing patients’ phenotypic characteristics, we categorized the patients into two distinct genotypic groups: deletion and non-deletion HbH disease subgroups. In the latter subgroup, we included patients with both non-deletion/non-deletion and the deletion/non-deletion arrangements together.

Statistical analysis was performed using Mircosoft® Excel, Professional Edition, 2010. Data was described in number and percentage. Quantitative data were described using mean, standard deviation median, and range. Chi-square test was used to compare categorical data. Mann-Whitney test was used to compare two sets of numerical data. Correlations between two quantitative variables were assessed using Spearman’s coefficient. Significance was considered at *P* < 0.05.

## Results

Over 6 years, 44 HbH disease patients, 19 males and 25 females, were recruited to this study. Their age ranged from 1 to 68 years, with a median of 19 years. The median Hb was 9.15 g/dL, ranging from 6.4 to 12.7. Table 1 shows some demographic and clinical parameters of the patients.

**Table 1:** Demographic and clinical characteristics of patients with HbH disease

Parameters	
Age, y (median, range)	19 (1–68)
Gender	
Male	19 (43.2%)
Female	25 (56.8%)
Hb, g/dL (median, range)	9.15 (6.4–12.7)
HbH, % (median, range)	7.35 (1–25.7)
Ferritin, ng/mL (median, range)	442 (11–2950)
Splenectomized	5 (11.4%)
Transfusion status	
Never transfused	23 (52.3%)
Occasional (≤1 unit per year)	17 (38.6%)
Irregular (2 to 4 units per year)	4 (9.1%)

**Fig. 1:**
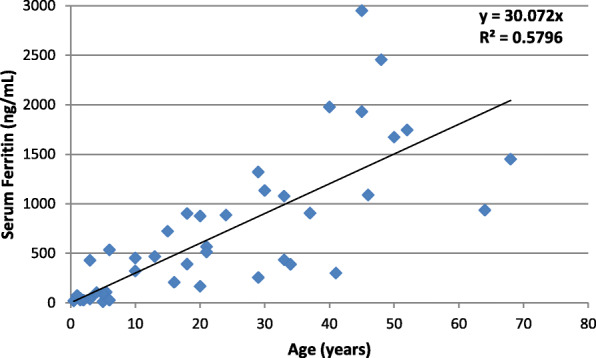
Correlation of serum ferritin with the age of HbH disease patients

Serum ferritin showed a positive correlation with age (*r* = 0.761) (Fig. 1). Splenectomized patients were 5 (11.4%), we found their mean age was noticeably higher compared to the non-splenectomized (34.6 versus 20.5 years respectively, *p* = 0.05). A significant relation (*p* < 0.001) was found between patients’ age and blood transfusion. There were 23 (52.3%) patients who never received a transfusion; their mean age was 10.5 years versus 35.4 years in the transfused patients. The Hb and ferritin levels were not different in the splenectomized and non-splenectomized patients (*p*-values 0.061 and 0.138, respectively). We also found significantly higher ferritin levels among transfused patients (*p* < 0.001).

The molecular characterization detected eight different α-globin mutations within the studied group. As illustrated in Table 2, −-^MED^ double-gene deletion was the most common mutation, which was observed in 33 (37.5%) alleles, followed by mutations of -α^3.7^ (35.2%), α2^poly-A1^ (15.9%), −α^4.2^ (3.4%), in addition to four other mutations which collectively comprised 8% of all mutated alleles. Totally, deletion mutations constituted 78.4% and non-deletion mutations 21.6% of all mutated alleles.

**Table 2:** Mutation frequency in HbH disease patients

Mutation	Type	Frequency	%
--^MED^	Deletion	33	37.5
-α^3.7^	Deletion	31	35.2
α2^poly-A1^ (AATAAA>AATAAG)	Point mutation	14	15.9
-α^4.2^	Deletion	3	3.4
-α^20.5^	Deletion	2	2.3
Codon 59 (GGC > GAC) (Hb Adana)	Point mutation	2	2.3
α2 IVS-I(−5 nt) (−TGAGG)	Splicing site 5 nt. Deletion	2	2.3
α2^poly-A2^ (AATAAA>AATGAA)	Point mutation	1	1.1
Total		88	100

The mutated alleles were found in nine different genetic arrangements. The most frequent genotype was --^MED^/−α^3.7^ arrangement, which was detected in 26 (59.1%) patients. The second most common genotype was homozygosity of α^Poly-A1^α, which was observed in 6 (13.6%) patients. Deletion genotypes we encountered in 31 patients (70.45%), while non-deletion genotypes were observed in 13 cases (29.54%). The α-globin genotypes and their relative frequencies are illustrated in Table 3.

**Table 3:** HbH disease genotypes

Genotypes	Frequency	%
--^MED^/−α^3.7^	26	59.1
Α^poly-A1^α/α^poly-A1^α	6	13.6
--^MED/^−α^4.2^	3	6.8
-α^20.5^/−α^3.7^	2	4.5
--^MED^/α^ivs1(−5 nt)^α	2	4.5
-α^3.7^/α^poly-A1^α	2	4.5
--^MED^/α^poly-A2^α	1	2.3
--^MED^/αα^Adana^	1	2.3
-α^3.7^/αα^Adana^	1	2.3
Total	44	100

Some relevant clinical and haematological parameters within the deletion and non-deletion HbH disease subgroups are illustrated in Table 4. The median values of the red cell count and Hb were higher in patients with deletion HbH disease; however, the differences were not statistically significant. All other haematological and clinical parameters did not show significant differences between the two groups.

**Table 4:** Clinical and haematological parameters in the deletion and non-deletion types HbH Disease

Parameters	Deletion (31 cases)	Non-Deletion (13 cases)	*p*-value
RBC count, (median, range)	5.05 (3.5–6.61)	4.51 (2.96–6.74)	0.069
Hb, g/dL (median, range)	9.2 (8.0–11.5)	8.3 (6.4–12.7)	0.086
MCV, fl (median, range)	61.3 (52.2–68.7)	62.6 (52.5–81.9)	0.309
MCH, pg (median, range)	18.0 (16.1–21.9)	18.9 (16.1–28)	0.241
RDW, % (median, range)	25.6 (16.9–30.7)	25.0 (18.7–36.3)	0.816
HbH, % (median, range)	7.5 (1–15)	7.2 (5.7–25.7)	0.125
Ferritin, ng/mL (median, range)	467 (11–2950)	389 (29–1726)	0.403
Splenectomized	4 (12.9%)	1 (7.7)	0.20
Transfusion			
Never transfused	16 (51.6%)	7 (53.8%)	0.57
Occasional (≤1 unit per year)	13 (41.9%)	4 (30.8%)	
Irregular (2 to 4 units per year)	2 (6.5%)	2 (15.4%)	

## Discussion

HbH disease is considered the most symptomatic form of α-thalassaemias that is compatible with life. The disease, however, has a heterogeneous clinical and haematological phenotype ranging from very mild phenotypes to more severe forms requiring regular or irregular blood transfusion. The severity of the disease is assumed to be related to the degree of α:β globin chain imbalance, which is principally determined by the nature of the underlying α-globin mutations [1, 10]. However, variations in the clinical phenotypes have been observed even in the presence of similar genotype, which is probably due to genetic and environmental modifiers [13].

Erbil province in northern Iraq covers an area of 15,075 km2 with a population of about two million people. Majority of the population are Kurds; the minorities of Turkmen, Assyrians, and Arabs comprise about 5% of its total population. The province is bordered by Turkey from the north-east and Iran from the east. We know much about β-thalassaemia in northern Iraq. Many studies have described the spectrum of β-globin gene mutations, the carrier rate, and the genotypic mechanisms in both thalassaemia major and thalassaemia intermedia [14–19]. Scrutinizing α-thalassaemia is not as easy as for β-thalassaemia because of the lower carrier rate in our region, and the difficulty in confirming diagnosis of α-thalassaemia carriers via the routine Hb-electrophoresis. The carrier rate of α-thalassaemia in Iraq is estimated at < 1%, but no study has yet rectified the exact carrier rate in this region. So far, a couple of small studies by Al-Allawi et al. have described the spectrum of α-globin gene mutations among carriers in the neighboring provinces of Dohuk and Sulaimaniyah [20, 21]. Results of the current study, which is the first to tackle a relatively large cohort of patients with HbH disease in this region, will be complementary to improve the existing national thalassemia carrier screening and genetic counseling program that was established in 2009, and will be helpful for prenatal diagnosis.

The clinical and haematological features of the enrolled patients showed obvious heterogeneity. Substantial variation was noticed in the patients’ age, Hb level, and transfusion requirement. Many clinical parameters strongly related to patients’ age, primarily the ferritin level and transfusion requirement. It is widely reported that the clinical severity of thalassaemias and other hemoglobinopathies is extremely age dependent [22].

In the current study, we have identified eight different α-globin gene mutations, four of which are deletion mutations (−-^MED^, −α^3.7^, −α^4.2^, and -α^20.5^) and the remaining four are non-deletion mutations (α2 poly-A1, Hb Adana, α2 IVS1 (− 5 nt), and α2 poly-A2). Comparable to the results of earlier reports by Al-Allawi et al. [20, 21], we found the spectrum of α-globin gene defects restricted to a relatively limited number of mutations. However, this finding is unlike to what have been observed in Iran, Turkey, Sardinia, and Cyprus, where the mutations are more heterogeneous [23, 24]. Within our HbH disease patients, the --^MED^ double-gene deletion was the most commonly encountered mutation (33 alleles) followed by the worldwide prevalent -α^3.7^ single gene deletion (31 alleles). Similar findings have been reported by the vast majority of studies done in the Mediterranean and Middle East regions [25]. The α2 poly-A1 non-deletion mutation, which is known to be prevalent in the Arabian Peninsula, was encountered in 14 alleles and was the most frequently encountered non-deletion mutation. Homozygosity for the latter mutation is the main genotype causing HbH disease in Saudi Arabia, UAE, Kuwait, and Bahrain [26–29]. None of the south-east Asian mutations were observed in this cohort. A previous study has reported the --^FIL^ double-gene deletion in one β-thalassaemia intermedia case who was homozygous for IVS-II-1 (G > A). The same deletion was also observed in Hatay Province of Turkey [18, 25].

We, in the current cohort, observed nine different genotypes causing HbH disease, most of which were deletion genotypes (70.45%, 31 patients). The --^MED^/−α^3.7^ arrangement was the most frequently encountered genotype as in the neighboring areas of Turkey and Iran. High prevalence of deletion type HbH disease have been reported in the Mediterranean area, Turkey, Iran, Greece, and Sardinia, (80, 50.5, 56.1, and 86% respectively) [10, 26, 30, 31]. We detected 19 alleles carrying non-deletion mutations of which 17 involved the α2 gene. Mutations of the α2-globin gene are associated with more severe phenotype because more than two-third of the α-globin chain synthesis is controlled by the dominant α2 globin gene transcription [32]. Hb Adana was the only detected α1 globin gene mutation in our patients; it was observed in compound heterozygosity with --^MED^ deletion in one patient and with -α^3.7^ deletion in another. We could not find any previous study reporting the latter combination (−α^3.7^/αα^Adana^) to have a HbH equivalent phenotype; though, we surprisingly found it in a 1.5 year-old boy with Hb of 7.1 g/dL. Coinheritance of -α^3.7^ deletion, with many other point mutations in the α2 globin gene, are reported in many studies [33]. In our cohort, coinheritance of two point mutations was detected in six patients (13.6%) only, who all were homozygous for α2 poly-A1 mutation. Non-deletion genotypes are reported to be the predominant HbH disease genotypes in the Arabia area, Jordan, and northern Iran [23, 27, 34, 35].

It has been previously documented that patients with deletion/deletion HbH disease genotype are of least severe phenotype, followed by deletion/non-deletion arrangement, and the non-deletion/non-deletion genotype which is supposed to be the most severe [6, 30]. In our cohort, we did not find any significant difference in the clinical and haematological parameters, which we studied in both deletion and non-deletion HbH disease subgroups. The median Hb and red cell count were notably lower in the non-deletion HbH subgroup, however not to a statistically significant level. The remaining red cell parameters, the ferritin level, the HbH fraction, splenectomy, and blood transfusion status did not show any difference in the two subgroups.

## Conclusion

This study showed that our HbH disease patients had variable clinical and haematological phenotypes ranging from very mild cases not requiring transfusion to more severe others needed irregular transfusions. The spectrum of α-globin gene mutations and then the underlying genotypic arrangements were not many. The majority of our HbH disease patients were found to have deletion genotypes; however, their clinical phenotypes did not differ much from those having non-deletion genotypes. Knowing all these will definitely have a positive impact on thalassaemia diagnosis and management guidelines in this region.

## Data Availability

The dataset used for the current study is available in the Mendeley datasets repository (DOI: 10.17632/bjgf3kcpgk.1). https://data.mendeley.com/datasets/bjgf3kcpgk/1

## Abbreviations

HbH: Hemoglobin-H
PCR: Polymerase chain reaction
HPLC: High performance liquid chromatography
Hb: Hemoglobin
RBCs: Red blood cells
MCV: Mean cell volume
MCH: Mean cell hemoglobin
RDW: Red cell distribution width
